# Map-based cloning and promoter variation analysis of the lobed leaf gene *BoLMI1a* in ornamental kale (*Brassica oleracea* L. var. *acephala*)

**DOI:** 10.1186/s12870-021-03223-y

**Published:** 2021-10-06

**Authors:** Bin Zhang, Wendi Chen, Xing Li, Wenjing Ren, Li Chen, Fengqing Han, Zhiyuan Fang, Limei Yang, Mu Zhuang, Honghao Lv, Yong Wang, Yangyong Zhang

**Affiliations:** Key Laboratory of Biology and Genetic Improvement of Horticultural Crops, Ministry of Agriculture, Institute of Vegetables and Flowers, Chinese Academy of Agricultural Sciences, #12 Zhong Guan Cun Nandajie Street, Beijing, 100081 China

**Keywords:** Ornamental kale, Lobed leaf, Fine mapping, Promoter variation, Enhanced expression

## Abstract

**Background:**

Leaf shape is an important agronomic trait in ornamental kale (*Brassica oleracea* L. var. *acephala*). Although some leaf shape-related genes have been reported in ornamental kale, the detailed mechanism underlying leaf shape formation is still unclear. Here, we report a lobed-leaf trait in ornamental kale, aiming to analyze its inheritance and identify the strong candidate gene.

**Results:**

Genetic analysis of F_2_ and BC_1_ populations demonstrate that the lobed-leaf trait in ornamental kale is controlled by a single dominant gene, termed *BoLl-1* (*Brassica oleracea* lobed-leaf). By performing whole-genome resequencing and linkage analyses, the *BoLl-1* gene was finely mapped to a 127-kb interval on chromosome C09 flanked by SNP markers SL4 and SL6, with genetic distances of 0.6 cM and 0.6 cM, respectively. Based on annotations of the genes within this interval, *Bo9g181710*, an orthologous gene of *LATE MERISTEM IDENTITY 1* (*LMI1*) in *Arabidopsis*, was predicted as the candidate for *BoLl-1*, and was renamed *BoLMI1a*. The expression level of *BoLMI1a* in lobed-leaf parent 18Q2513 was significantly higher compared with unlobed-leaf parent 18Q2515. Sequence analysis of the parental alleles revealed no sequence variations in the coding sequence of *BoLMI1a*, whereas a 1737-bp deletion, a 92-bp insertion and an SNP were identified within the *BoLMI1a* promoter region of parent 18Q2513. Verification analyses with *BoLMI1a*-specific markers corresponding to the promoter variations revealed that the variations were present only in the lobed-leaf ornamental kale inbred lines.

**Conclusions:**

This study identified a lobed-leaf gene *BoLMI1a*, which was fine-mapped to a 127-kb fragment. Three variations were identified in the promoter region of *BoLMI1a*. The transcription level of *BoLMI1a* between the two parents exhibited great difference, providing new insight into the molecular mechanism underlying leaf shape formation in ornamental kale.

**Supplementary Information:**

The online version contains supplementary material available at 10.1186/s12870-021-03223-y.

## Background

Leaves are essential organs that play an important role in plants, including carbon assimilation, gas exchange, water transport and nutrient distribution [1]. Leaf shape can significantly affect both leaf function and plant architecture [2, 3]. A typical variation in leaf shape involves the leaf margin, which can be unlobed, serrated or lobed [4]. Lobed leaves can be easily visualized even in the primary leaf stage, which can be used as an indicator trait for hybrid production [5, 6]. Compared to unlobed- or serrated-leaf lines, plants with lobed leaves are better adapted to environmental stresses [7, 8]. With improved heat transfer and light energy absorption, lobed leaves are advantageous for high-density planting and mechanized production [9]. Additionally, lobed leaves are also a graceful decorative trait for ornamental plants such as kale [4].

Ornamental kale (*Brassica oleracea* L. var. *acephala*) is an attractive ornamental crop owing to its polymorphic, colorful leaves [10]. Lobed-leaf genes have been genetically analyzed and mapped in some *Brassica* species. For example, the lobed-leaf trait in *B. rapa* is controlled by major gene or polygenic effects [11–14]. In *B. napus*, the incomplete dominant lobed-leaf gene *BnLL1* was mapped to the distal end of chromosome A10 [15]. In ornamental kale, some studies have shown that the lobed-leaf trait exhibits incomplete dominance over the unlobed-leaf trait [16]. Genetic analysis of an interspecific hybrid between *B. napus* and *Rorippa indica* (L.) Hiern revealed that the lobed-leaf trait is controlled by a dominant gene [9]. Moreover, Ren et al. mapped a quantitative trait locus (QTL) associated with lobed leaves to chromosome 9 of ornamental kale flanked by insertion-deletion (InDel) markers LYIn39 and LYIn40, with genetic distances of 0.17 cM and 0.11 cM, respectively [4].

With the development of high-throughput sequencing technology and the release of *B. oleracea* draft genomes [17, 18], a growing number of genes that govern important traits have been mapped in this species. Bulk segregant analysis (BSA) is a rapid and accurate gene mapping method that was first developed and performed in plants [19]. This method is characterized by bulk genotyping of a pool of segregants that share the same phenotype. InDel has been considered as an ideal source for marker design due to its high-density distribution and genotyping efficiency. Using InDel markers, many genes/QTLs have been mapped in *B. oleracea*, including the yellow-green leaf gene *ygl-1* [20], the purple leaf gene *BoPr* [21], QTLs associated with heading traits [22], male sterility genes [23, 24] and the petal color gene *BoCCD4* [25].

Lobed-leaf trait is a unique variation in kale that can be produced by infrequent genetic mechanisms. In the present study, we developed F_1_, F_2_ and BC_1_ populations descended from the ornamental kale inbred line 18Q2513 (with lobed leaves) and 18Q2515 (with unlobed leaves). A rare dominant inheritance pattern was identified for lobed-leaf trait using these populations. Furthermore, the lobed-leaf gene *BoLl-1* was fine-mapped to a narrow interval using BSA-seq and linkage analysis. The findings provide new insight into the molecular mechanism underlying leaf shape formation in ornamental kale.

## Results

### Genetic analysis of leaf shape in ornamental kale

**Fig. 1:**
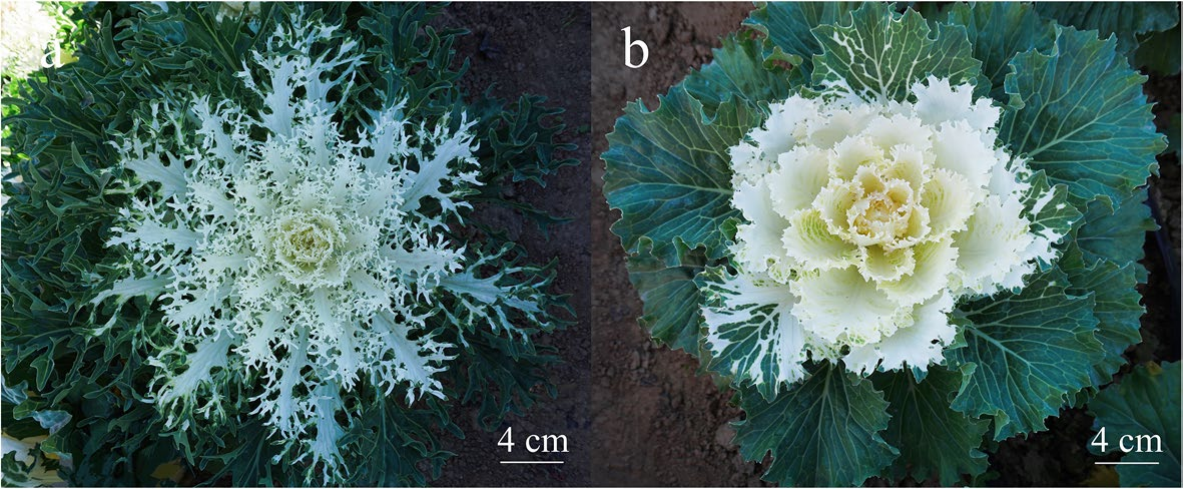
Leaf phenotypes of the parental lines. **a** 18Q2513 with lobed leaves. **b** 18Q2515 with unlobed leaves. Bar = 4 cm

The leaf shape throughout all the F_1_ plants (comprising 16 individuals) generated by crossing 18Q2513 (lobed-leaf, Fig. 1a) with 18Q2515 (unlobed-leaf, Fig. 1b) was lobed; thus, the lobed-leaf trait is dominant over the unlobed-leaf trait in these two ornamental kale lines. The F_2_ population comprised 120 individuals, with 92 displaying lobed leaves and 28 unlobed leaves. According to a chi-square test, the segregation ratio is 3:1. The BC_1_P_1_ population contained 850 individuals, with 429 lobed-leaf individuals and 421 unlobed-leaf individuals, and the segregation ratio was confirmed to be 1:1 by a chi-square test. The 200 BC_1_P_2_ individuals all had lobed leaves (Table 1). These results indicate that the lobed-leaf trait is controlled by a single dominant gene, which was named *BoLl-1*.

**Table 1:** The Chi-square (χ^2^) goodness-of-fit test ratios of leaf shape segregation in BC and F_2_ populations

Populations	Total plant number	Number of lobed-leaf individuals*	Number of unlobed-leaf individuals*	Expected ratio	χ^2a^
F_1_	16	16	0	–	–
F_2_	120	92	28	3:1	0.18
BC_1_P_1_	850	429	421	1:1	0.08
BC_1_P_2_	200	200	0	–	–

^a^χ^2^ > χ^2^_0.05_ = 3.84 was considered significant

*Lobed-leaf plants and unlobed-leaf plants were determined at the seedling stage by visual inspection

### Fine mapping of the *BoLl-1* gene by BSA-seq and linkage analyses

**Fig. 2:**
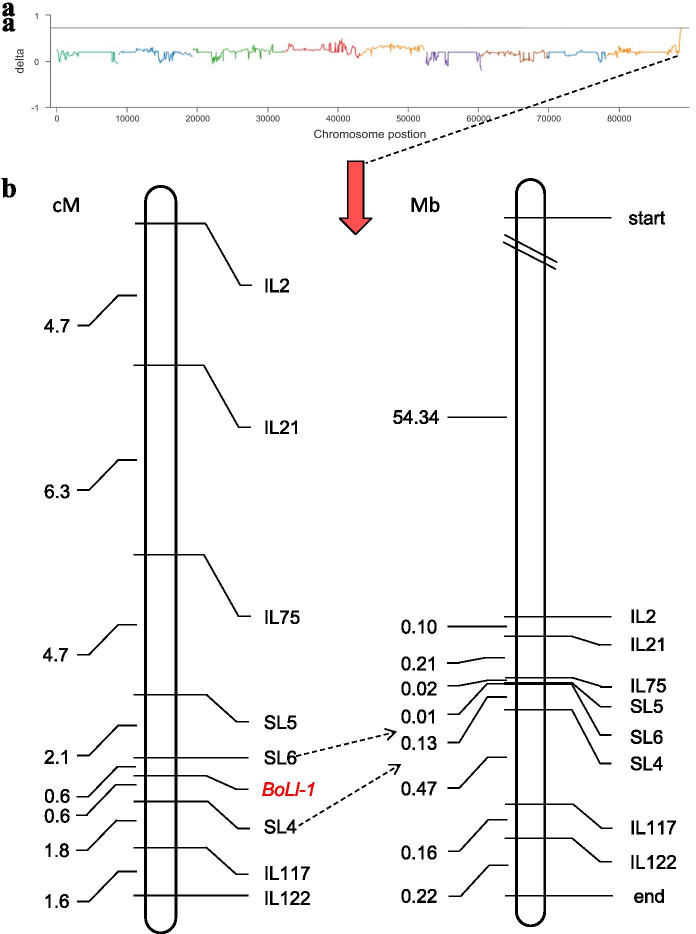
Fine mapping of the *BoLl-1* gene in ornamental kale. **a** Plot of the Δ(SNP-index) value obtained from the two bulks. The top line indicates the threshold line. The x-axis represents the position of nine chromosomes and the y-axis represents the Δ(SNP-index). **b** Linkage map of the *BoLl-1.* The left panel is a genetic map of *BoLl-1* in the target region (units: cM). The right panel is the corresponding physical map of *BoLl-1* (units: Mb)

To identify markers associated with lobed leaves, the SNP index and Δ(SNP index) between the two bulks were calculated using high-quality SNPs. The average SNP-index and Δ(SNP-index) of the two bulks across a 1-Mb genomic interval were measured using a 10-kb sliding window and plotted against the genome position. The highest peak region, which was considered to be the candidate interval associated with *BoLl-1*, contains approximately 1.33 Mb (53.34–54.67 Mb) on chromosome 9 according to the ‘TO1000’ reference genome (Fig. 2a). For the candidate region of *BoLl-1*, 3280 SNPs between parental lines were identified, 410 of which are effective; 593 InDels were identified, 35 of which are effective (Table S1).

To further delineate the location of *BoLl-1*, 16 InDel and seven SNP markers (by comparing resequencing data of the parents with the sequence of the TO1000 reference genome) within the 1.33-Mb candidate region and its flanking regions (600 kb on each side) were designed. Ultimately, five InDel and three SNP markers showed polymorphisms between the two parents. A total of 429 recessive individuals of the BC_1_P_1_ population were subsequently used for *BoLl-1* fine mapping.

A linkage map consisting of five InDel and three SNP markers was constructed using MapDraw (Fig. 2b). The SNP markers SL4 and SL6 were found to be tightly linked to *BoLl-1*, with genetic distances of 0.6 cM and 0.6 cM, respectively. Based on the marker locations in the reference genome, *BoLl-1* was ultimately delimited to a 127-kb region (53680797–53,808,289 bp) on chromosome C09.

### Prediction and expression analysis of the candidate genes

Based on the ‘TO1000’ reference genome [18], 21 genes were identified within the 127-kb interval (Table 2). According to annotations from the *Brassica oleracea* genome and BLASTX (best hit) to *A. thaliana*, only two genes *Bo9g181710* and *Bo9g181720* are related to the formation of leaf shape. These two genes are homologues of the *LATE MERISTEM IDENTITY 1* (*LMI1*) gene in *Arabidopsis,* which encode a homeodomain leucine zipper class I (HD-Zip I) meristem identity regulator that plays an important role in leaf morphogenesis and bract formation. Thus, we designated that *Bo9g181710* and *Bo9g181720* were candidate genes controlling lobed leaf shape in ornamental kale.

**Table 2:** The 21 putative gene models in the target mapping region

Gene ID	Location	Homologous gene in *A. thaliana*	Annotation
*Bo9g181620*	C9: 53678143–53,680,993	*AT5G03900*	iron-sulphur cluster biosynthesis family protein
*Bo9g181630*	C9: 53681760–53,682,275	*AT5G03890*	hypothetical protein
*Bo9g181640*	C9: 53685355–53,687,538	*AT5G03880*	thioredoxin family protein
*Bo9g181650*	C9: 53697594–53,698,572	*–*	–
*Bo9g181660*	C9: 53698893–53,699,987	*AT5G03850*	nucleic acid-binding, OB-fold-like protein
*Bo9g181670*	C9: 53703655–53,704,734	*AT5G03840*	protein TERMINAL FLOWER 1
*Bo9g181680*	C9: 53711576–53,712,658	*–*	–
*Bo9g181690*	C9: 53713320–53,715,156	*AT5G03795*	probable glycosyltransferase
*Bo9g181700*	C9: 53717227–53,718,573	*AT5G03795*	probable glycosyltransferase
*Bo9g181710*	C9: 53720142–53,721,856	*AT5G03790*	encodes a homeodomain leucine zipper class I (HD-Zip I) meristem identity regulator
*Bo9g181720*	C9: 53749509–53,750,894	*AT5G03790*	encodes a homeodomain leucine zipper class I (HD-Zip I) meristem identity regulator
*Bo9g181730*	C9: 53755444–53,758,253	*AT5G03770*	probable 3-deoxy-D-manno-octulosonic acid transferase
*Bo9g181740*	C9: 53760949–53,762,178	*–*	–
*Bo9g181750*	C9: 53765687–53,769,942	*AT5G03760*	glucomannan 4-beta-mannosyltransferase 9
*Bo9g181760*	C9: 53771597–53,773,283	*AT5G03740*	histone deacetylase 2C
*Bo9g181770*	C9: 53777463–53,782,597	*AT5G03730*	serine/threonine-protein kinase CTR1
*Bo9g181780*	C9: 53783895–53,785,735	*AT5G03720*	heat shock transcription factor A3
*Bo9g181790*	C9: 53793205–53,794,037	*–*	–
*Bo9g181800*	C9: 53801111–53,802,559	*AT5G03700*	D-mannose binding lectin protein with Apple-like carbohydrate-binding domain
*Bo9g181810*	C9: 53803210–53,804,612	*AT5G03690*	fructose-bisphosphate aldolase 4
*Bo9g181820*	C9: 53807596–53,810,446	*AT5G03680*	trihelix transcription factor PTL

**Fig. 3:**
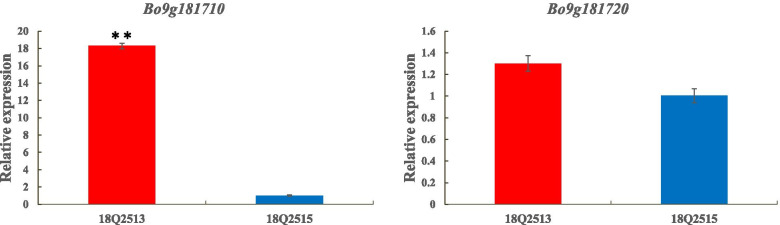
Expression patterns of *Bo9g181710* and *Bo9g181720* as determined by qRT-PCR between 18Q2513 and 18Q2515. *BoActin* served as the equal loading control. The error bars represent standard errors of three biological replicates. Asterisks represent significant differences (*p* < 0.01)

To analyze the expression patterns of *Bo9g181710* and *Bo9g181720*, qRT-PCR was performed using young leaves from 28-day-old seedling of the parents. The expression level of *Bo9g181710* in lobed-leaf 18Q2513 was significantly higher than that in unlobed-leaf 18Q2515, whereas no significant difference in *Bo9g181720* expression between the parental lines was detected (Fig. 3).

### Sequence analysis of the candidate genes

**Fig. 4:**
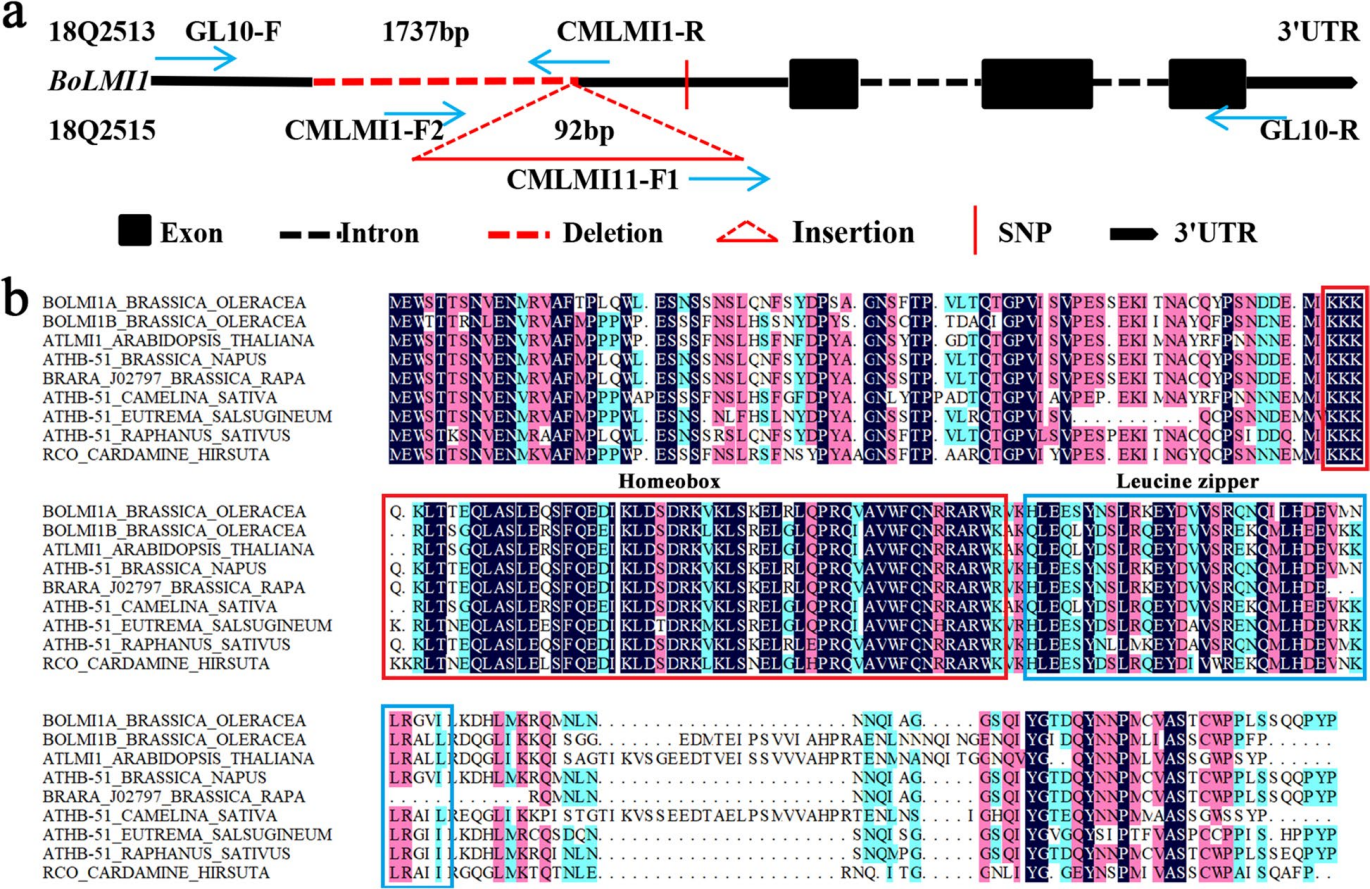
Gene structure and protein alignment of *BoLMI1a*. **a** The *BoLMI1a* gene structure as well as promoter variations between 18Q2513 and 18Q2515 are shown; horizontal blue arrows represent specific primers for amplifying the promoter and genomic sequences and detecting the promoter variations of *BoLMI1a*. **b** Sequence alignment of the *BoLMI1a* and *BoLMI1b* proteins and its seven homologues from other cruciferous species. The homeobox domain as well as the leucine zipper domain are indicated

To determine the causal relationship between the candidate genes and leaf shape formation, a comparative sequence analysis of the *Bo9g181710* and *Bo9g181720* genes body (DNA) and ~ 3-kb promoter region was performed using genomic DNA from 18Q2513 and 18Q2515. No sequence variations (between the parental lines) in the coding sequences of *Bo9g181710* were detected, while a 1737-bp deletion (1466 bp upstream of the transcription start site), a 92-bp insertion (1466 bp upstream of the transcription start site) and an SNP (765 bp upstream of the transcription start site) were identified within the *Bo9g181710* promoter region of 18Q2513 (Fig. 4a). Conversely, no variation was detected in either the promoter or coding regions of *Bo9g181720* between the 18Q2513 and 18Q2515. Combined with expression analysis, we speculated that the *Bo9g181710* may control the formation of leaf shape in ornamental kale, and renamed it *BoLMI1a*. In addition, we renamed the *Bo9g181720* to *BoLMI1b*.

**Fig. 5:**
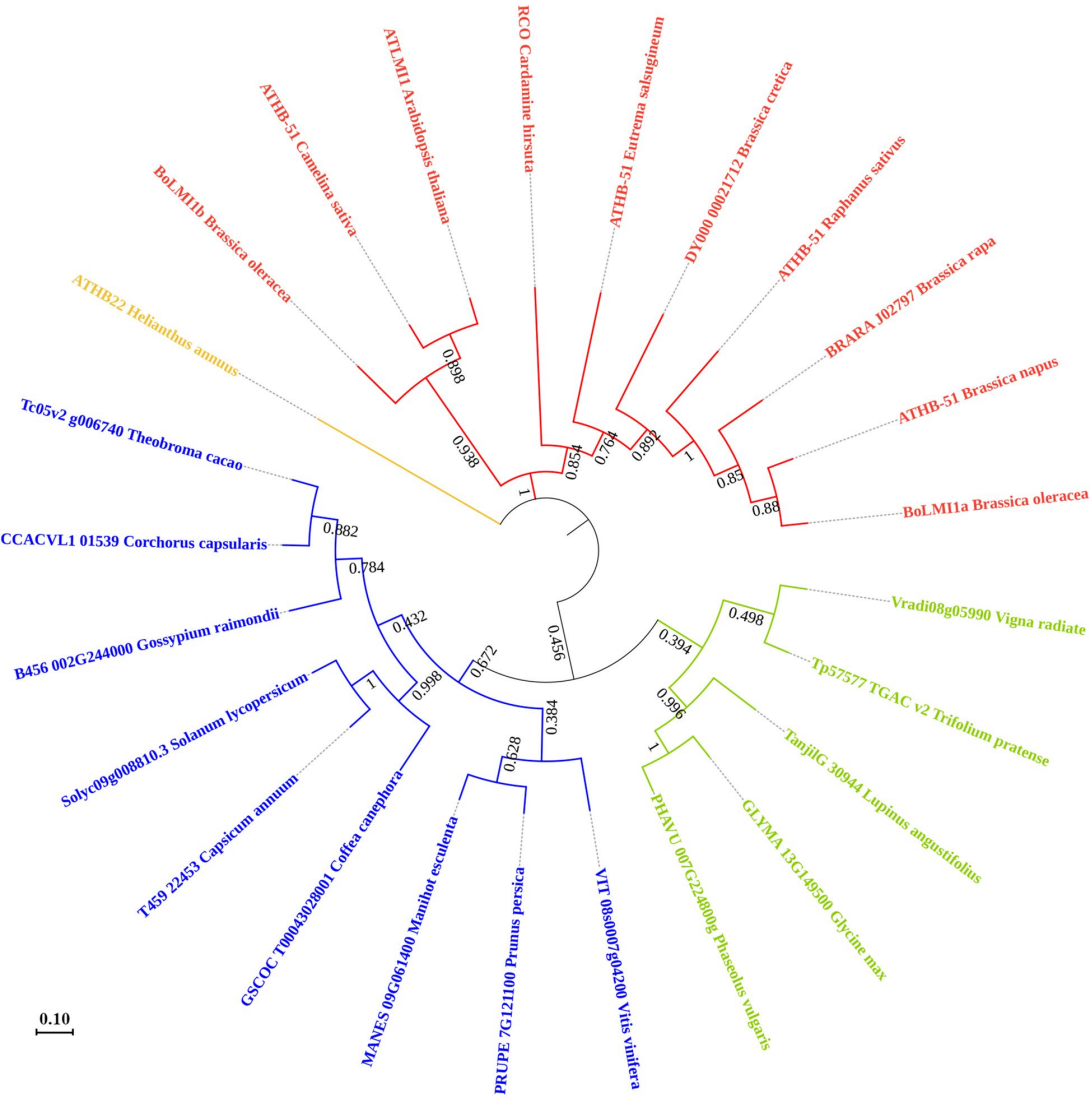
Phylogenetic analysis of *BoLMI1a* and *BoLMI1b* proteins and its 23 homologues from other species. Numbers are bootstrap values

Sequence analysis further revealed that *BoLMI1a*, which consists of three exons and two introns, encodes a putative 219-amino acid protein containing a homeobox domain and a leucine zipper domain (Fig. 4). Sequence alignment of the *BoLMI1a* and *BoLMI1b* proteins and its seven homologues from other cruciferous species revealed that *BoLMI1a* shared a high degree of similarity with its homologues in *B. napus* (98.17%) and *B. rapa* (91.32%) but a relatively lower degree of similarity with *BoLMI1b* (59.41%) and *Camelina sativa* (54.92%) (Fig. 4b). Furthermore, a phylogenetic analysis of the *BoLMI1a* and *BoLMI1b* proteins and its close homologues was carried out to evaluate their evolutionary relatedness. The results showed that *BoLMI1a* is closely related to *B. napus ATHB-51* and is located in the same clade as other cruciferous plants, indicating that they may be derived from the same ancestor gene (Fig. 5).

### Verification of *BoLMI1a*-specific markers

**Fig. 6:**
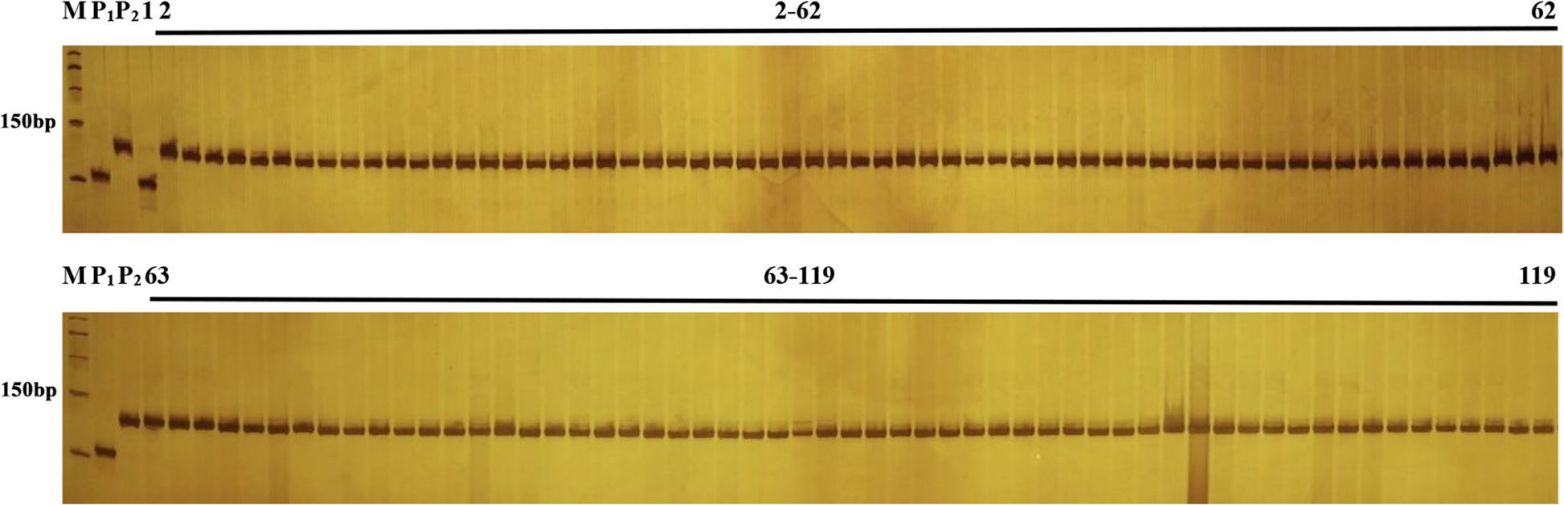
Amplicons of the co-dominant marker CMLMI1 in parents and 118 different cabbage inbred lines and ornamental kale inbred lines 18Q2523. M represents the DNA ladder, P_1_ is lobed-leaf inbred line 18Q2513, and P_2_ is unlobed-leaf inbred line 18Q2515. Lane 1 is ornamental kale inbred line 18Q2523 with lobed leaves, Lanes 2–119 are 118 different cabbage inbred lines with unlobed leaves

Using the co-dominant marker CMLMI1 and the dCAPS marker DMLMI1, we determined whether the variations the *BoLMI1a* promoter are also present in 118 different cabbage inbred lines (with unlobed leaves) and another ornamental kale inbred line 18Q2523 (with lobed leaves). The results indicated that the insertion, deletion (detected by co-dominant marker CMLMI1) and the SNP (detected by dCAPS marker DMLMI1) were present only in the lobed-leaf ornamental kale inbred line 18Q2523 (Fig. 6; Fig. S1). These markers exhibited 100% accuracy which can be used for marker-assisted selection. Overall, the analyses strongly indicated that the variations in the promoter of *BoLMI1a* exist only in lobed-leaf ornamental kale inbred lines and may be responsible for the change in leaf shape from unlobed to lobed.

## Discussion

In previous studies, the lobed-leaf trait was reported to be controlled by an incomplete gene or a QTL in ornamental kale [4, 16, 26]. In the present study, we analyzed the inheritance of leaf shape using F_2_ and BC populations derived from a cross of lobed-leaf ornamental kale with unlobed-leaf ornamental kale, showing that the lobed-leaf trait is controlled by a single dominant nuclear-encoded gene.

Ren et al. mapped the lobed-leaf gene *BoLl* to chromosome 9 of ornamental kale flanked by InDel markers LYIn39 and LYIn40, with genetic distances of 0.17 cM and 0.11 cM, respectively [4]. Two candidate genes, *Bol010029*/*Bo9g181710* and *Bol010030*/*Bo9g1181720,* were revealed, but no sequence variations were found in their promoter and coding regions according to the *B. oleracea* ‘02–12’ (cabbage) [17] and ‘TO1000’ (Chinese kale like) genomes [18]. Therefore, the authors did not conclude which gene controlled the formation of leaf shape in ornamental kale. In our study, based on the ‘TO1000’ genome, the *BoLl-1* gene was finely mapped to a 127-kb (53680797–53,808,289 bp) interval on chromosome 9. SNP markers SL4 and SL6 were tightly linked to *BoLl-1*, flanking the gene at genetic distances of 0.6 cM and 0.6 cM, respectively. Sequence analysis of the parental alleles revealed no sequence variations in the coding sequence of *Bo9g181710*, whereas three variations were identified in the promoter region. In contrast, no sequence variations were detected in the promoter and coding regions of *Bo9g181720*. The expression level of *Bo9g181710* in lobed-leaf 18Q2513 was significantly higher compared with unlobed-leaf 18Q2515, though the expression level of *Bo9g181720* was similar between the parental lines. Thus, we further confirmed that the *Bo9g181710* may control the formation of leaf shape in ornamental kale.

In *B. napus*, Hu et al. reported that a 2624-bp insertion (317 bp upstream of the transcription start site) and three SNPs were identified in the *BnA10.LMI1* promoter sequence, along with 12 SNPs in the 3′ flanking sequence, which were considered to be the cause of the lobed-leaf formation [27]. In ornamental kale, the genes that determine leaf shape are not fully understood. Ren et al. mapped the *BoLl* gene and found no sequence variations in the promoter and coding regions of candidate [4]. In our study, three variations, including an SNP, a 1737-bp deletion, and a 92-bp insertion (765 bp, 1466 bp, and 1466 bp upstream of the transcription start site, respectively) were identified in the *BoLMI1a* promoter region compared with the ‘TO1000’ reference genome. Through verification analyses of *BoLMI1a*-specific markers corresponding to the promoter variations revealed that the variations existed only in lobed-leaf ornamental kale inbred lines. These variations may strongly enhance the transcription levels of *BoLMI1*, thus changing leaf shape from unlobed to lobed.

Leaf shape plays an important role in the reproduction and evolution of plants. Increasing evidence indicates that lobed leaves can improve photosynthesis efficiency and agronomic profitability [7, 28–31]. *LMI1*-like genes encoding an HD-Zip I transcription factor have been functionally identified in several plants, and they were reportedly involved in leaf shape formation [27, 32–35]. For example, Hu et al. [27] identified the *BnA10.LMI1* gene, which was responsible for the lobed-leaf shape in *Brassica napus*. In addition, the *BnA10.LMI1* knockout mutations in the HY (with lobed leaves) background were sufficient to produce unlobed leaves. In this study, we identified an *LMI1*-like gene, *BoLMI1a*, which was the strong candidate gene underlying the lobed-leaf trait in ornamental kale. Thus, our findings further strengthen the potential for revealing the molecular mechanism underlying leaf shape formation, and we showed that *BoLMI1a*-specific markers (CMLMI1 and DMLMI1) can be used for marker-assisted selection in ornamental kale breeding.

## Conclusions

In this study, the lobed-leaf trait is shown to be controlled by a single dominant gene, *BoLl-1*, in ornamental kale. The *BoLl-1* gene was fine-mapped to a 127-kb fragment. A homologue of *Arabidopsis LMI1*, *BoLMI1a* was identified as a strong candidate gene. Three variations were identified in the promoter region of *BoLMI1a.* The expression of *BoLMI1a* in lobed-leaf parent 18Q2513 was significantly up-regulated compared with unlobed-leaf parent 18Q2515. This study lays a foundation for cloning *BoLMI1a* and provides new insight into the formation of leaf shape in ornamental kale.

## Methods

### Plant materials

The 18Q2513 female parent (P_1_) is an ornamental kale inbred line with lobed leaves; the 18Q2515 male parent (P_2_) is an ornamental kale inbred line with unlobed leaves. 18Q2513 was crossed with 18Q2515 to generate an F_1_ population. An F_2_ population was generated from self-pollination of the F_1_ plants; BC_1_P_1_ and BC_1_P_2_ were then generated by BCs of F_1_ × 18Q2513, F_1_ × 18Q2515, respectively.

Additionally, 118 different cabbage inbred lines (with unlobed leaves) and another ornamental kale inbred line 18Q2523 (with lobed leaves), were screened for *BoLl-1* promoter variations. All of the plant materials used in the present study were grown in a 25 °C ± 2 °C greenhouse (16 h light/8 h dark photoperiod) at the seedling stage and then transplanted to the field after 1 month. Daily watering and fertilization were performed regularly until the plants enter the flowering stage (about 3 months of vernalization from December to February of the next year). All the plant materials are from the Institute of Vegetables and Flowers, Chinese Academy of Agriculture Sciences (IVFCAAS, Beijing, China).

### Genetic analysis and whole-genome resequencing

Leaf shape was investigated visually. Segregation ratios for the F_2_ and BC_1_ populations were analyzed by chi-square (χ^2^) tests using SAS software.

Fifty lobed-leaf BC_1_ and fifty unlobed-leaf BC_1_ individuals were selected to construct two bulks. Genomic DNAs were isolated from the individuals within the two bulks and two parental lines using the Plant Genomic DNA Kit (Tiangen, Beijing, China), following the manufacturer’s instructions. The quality of the DNAs was ensured using spectrophotometric analysis and agarose gel electrophoresis. Equally high-quality genomic DNAs from the two bulks and two parental lines were then used to construct paired-end sequencing libraries, which were subsequently sequenced with an Illumina Hi-Seq 2500 sequencer by the Beijing Genomics Institute (BGI) (Shenzhen, China). SNP-index and sliding-window analyses were performed as previously described [36].

### Marker development and fine mapping of the *BoLl-1* gene

InDel and SNP markers were designed based on candidate region resequencing data for the two parents. Markers were designed with amplicon lengths of 100–180 bp, GC contents of 40–50% and Tm values of 52–58 °C. The markers that were polymorphic between the parents were then used to analyze unlobed-leaf individuals in the BC_1_P_1_ populations.

Genomic DNA was extracted from 28-day-old seedling young leaves of the parents and BC_1_P_1_ individuals using a modified cetyltrimethylammonium bromide (CTAB) protocol [37]. The DNA concentration was subsequently determined using a spectrophotometer (BioDrop, UK) and adjusted to 40–50 ng/μL.

The 10-μL PCR reaction mixture consisted of 2 μL DNA template, 1 μL 10× PCR buffer (Mg^2+^ included), 0.8 μL dNTPs (2.5 mM each), 0.4 μL forward primer (10 μM), 0.4 μL reverse primer (10 μM), 0.2 μL Taq DNA polymerase (5 U/μL), and 5.2 μL ddH_2_O. The reactions were performed in accordance with the follows: 94 °C for 5 min, followed by 35 cycles of 94 °C for 30 s, 56 °C for 30 s and 72 °C for 45 s; and then 72 °C for 10 min. The amplicons were separated by 8% polyacrylamide gel electrophoresis (160 V for 1.2 h), and the gel was stained with silver nitrate.

For each marker, individuals consistent with the 18Q2513 (lobed-leaf) allele, the 18Q2515 (unlobed-leaf) allele, and the F_1_ allele were categorized as ‘a’, ‘b’, and ‘h’, respectively. Genetic distances between markers were calculated by the Kosambi map function [38], and a genetic map was constructed using MapDraw [39].

### Candidate gene analysis

To identify the lobed-leaf gene *BoLl-1*, genes located within the candidate interval were analyzed based on annotations for the *B. oleracea* ‘TO1000’ reference genome (http://plants.ensembl.org/Brassica_oleracea/Info/Index) [18]. The expression patterns of candidate genes *Bo9g181710* and *Bo9g181720* were investigated using quantitative real-time PCR (qRT-PCR). Total RNA was extracted from 28-day-old seedling young leaves of the parents using TRIzol reagent (Invitrogen, United States) according to the manufacturer’s protocol, and PrimeScript™ RT Reagent Kit (Takara, Japan) was used to reverse transcribe cDNA from the total RNA extracted. qRT-PCR was carried out using a CFX96 Real-Time System (Bio-Rad) with SYBR Premix Ex TaqII Reagent Kit (Takara, Japan). Three biological and three technical replicates were included for each experiment. The relative expression level of each gene was calculated using the 2^−ΔΔCt^ method [40]. The qRT-PCR primers used are listed in Table S2, and *B. oleracea actin* was employed as a control.

Gene-specific markers GL10 (primers GL10-F and GL10-R) and GL20 (primers GL20-F and GL20-R) (Table S2) were used to amplify the promoter and genomic sequences of *Bo9g181710* and *Bo9g181720*, respectively. The resulting PCR products were analyzed by electrophoresis on 1% agarose gels, followed by sequencing and alignment. The co-dominant marker CMLMI1 (primers CMLMI1-F1, CMLMI1-F2 and CMLMI1-R) and the derived cleaved amplified polymorphic sequence (dCAPS) marker DMLMI1 (primers DMLMI1-F and DMLMI1-R) (Table S2) were used to detect variations in the promoter of *BoLMI1a* in 118 different cabbage and ornamental kale inbred lines.

BLASTP searches were conducted using the amino acid sequence of *BoLMI1a* to search for homologues within the protein database of the National Center for Biotechnology Information (NCBI) and the *B. oleracea* reference genome ‘TO1000’. Protein sequence alignment was performed with MAFFT (v7.037) [41]. FastTree (LG + JTT model) was used to construct phylogenetic trees [42].

## Supplementary Information

**Additional file 1: Table S1.** Details of SNP and InDel variations in the candidate region of *BoLl-1*.

**Additional file 2: Table S2.** Primer sequences of the markers used in this study.

**Additional file 3: Figure S1.** Amplicons of the dCAPS marker DMLMI1 in parents and 118 different cabbage inbred lines and ornamental kale inbred lines 18Q2523. M represents the DNA ladder, P_1_ is lobed-leaf inbred line 18Q2513, and P_2_ is unlobed-leaf inbred line 18Q2515. Lane 1 is the ornamental kale inbred line 18Q2523 with lobed leaves, Lanes 2–119 are 118 different cabbage inbred lines with unlobed leaves.

**Additional file 4: Figure S2.** The original, full-length gel and blot images of Fig. 6. **Figure S3.** The original, full-length gel and blot images of Fig. S1.

## Data Availability

All data generated or analyzed during this study are included in this published article and its supplementary information files. The raw sequencing data used during this study are available in the NCBI SRA database (Accession number: PRJNA729727, https://www.ncbi.nlm.nih.gov/sra/PRJNA729727). The *B. oleracea* reference genome ‘TO1000’ used in this study can be found at the link: http://plants.ensembl.org/Brassica_oleracea/Info/Index. The *A. thaliana* genome can be found at the link: https://www.arabidopsis.org/. The protein database of National Center for Biotechnology Information (NCBI) can be found at the link: https://www.ncbi.nlm.nih.gov/. All these databases are open to public access.

## Abbreviations

BC: Backcross
SNP: Single Nucleotide Polymorphism
*LMI1*: *LATE MERISTEM IDENTITY 1*
QTL: Quantitative Trait Locus
InDel: Insertion/Deletion
BSA: Bulk Segregant Analysis
dCAPS: Derived Cleaved Amplified Polymorphic Sequences
NCBI: National Center for Biotechnology Information
