# Supplementary figures and images for: Map-based cloning and promoter variation analysis of the lobed leaf gene BoLMI1a in ornamental kale (Brassica oleracea L. var. acephala)
Source: BMC Plant Biol. 2021 Oct 6;21:456. doi: 10.1186/s12870-021-03223-y (PMC8496080; doi:10.1186/s12870-021-03223-y)

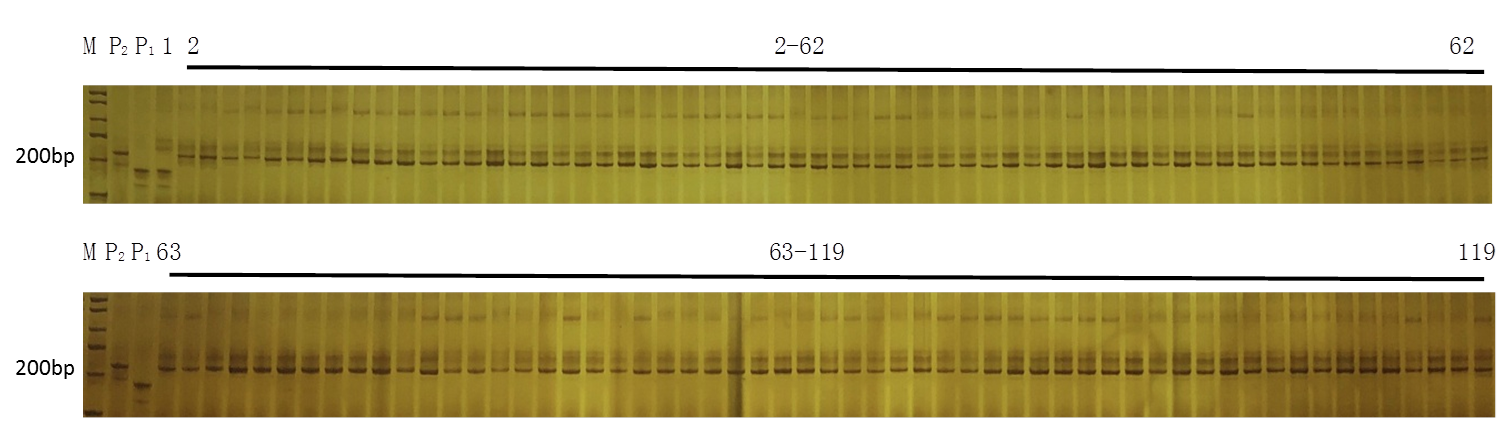

Supplement: Supplementary file 3 — Additional file 3: Figure S1. Amplicons of the dCAPS marker DMLMI1 in parents and 118 different cabbage inbred lines and ornamental kale inbred lines 18Q2523. M represents the DNA ladder, P1 is lobed-leaf inbred line 18Q2513, and P2 is unlobed-leaf inbred line 18Q2515. Lane 1 is the ornamental kale inbred line 18Q2523 with lobed leaves, Lanes 2–119 are 118 different cabbage inbred lines with unlobed leaves. [file 12870_2021_3223_MOESM3_ESM.png]
