# Supplementary material for: Map-based cloning and promoter variation analysis of the lobed leaf gene BoLMI1a in ornamental kale (Brassica oleracea L. var. acephala)
Source: BMC Plant Biol. 2021 Oct 6;21:456. doi: 10.1186/s12870-021-03223-y (PMC8496080; doi:10.1186/s12870-021-03223-y)
Supplement: Supplementary file 4 — Additional file 4: Figure S2. The original, full-length gel and blot images of Fig. 6. Figure S3. The original, full-length gel and blot images of Fig. S1. [file 12870_2021_3223_MOESM4_ESM.docx]

All original, full-length gel and blot images.


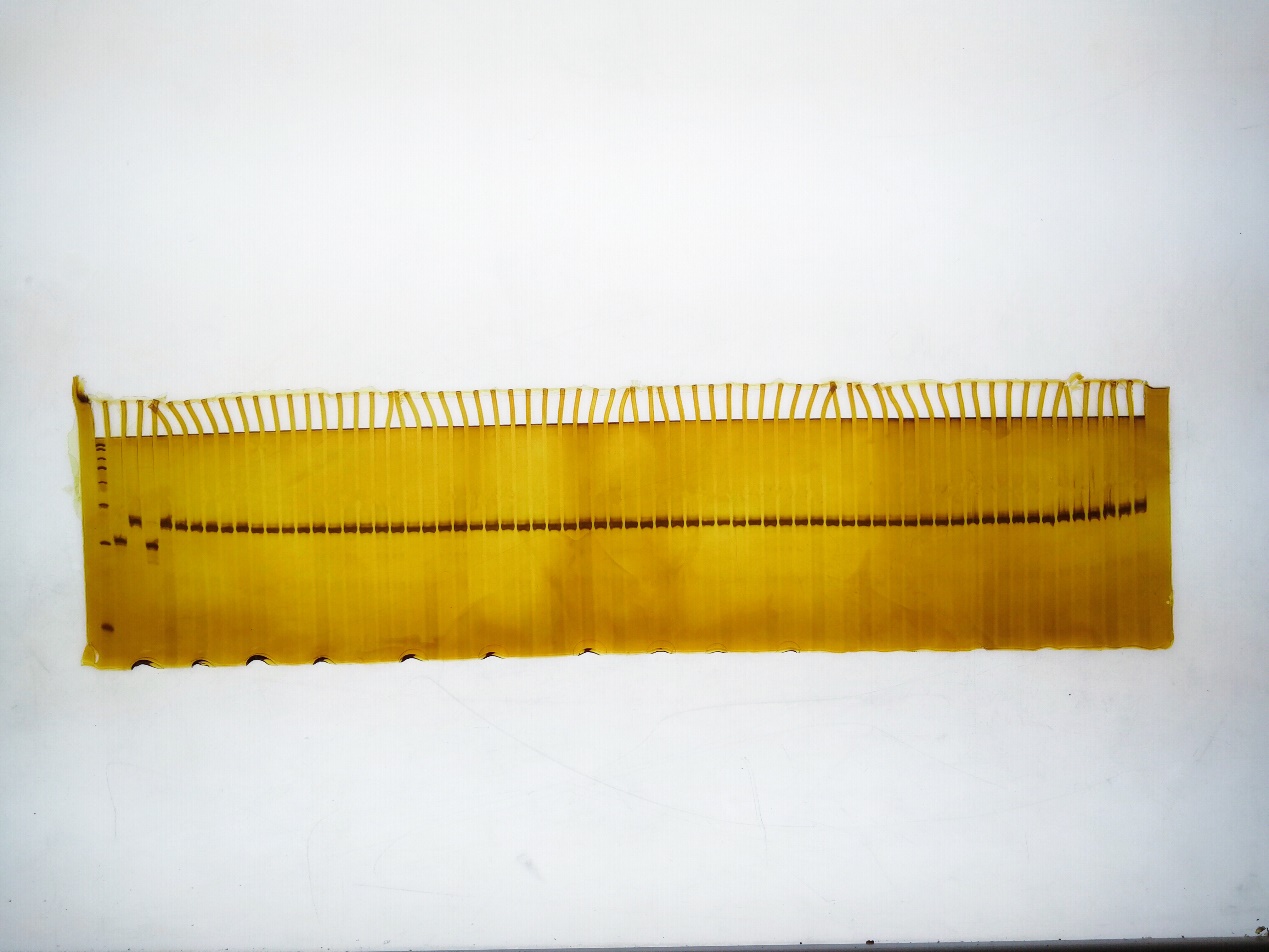


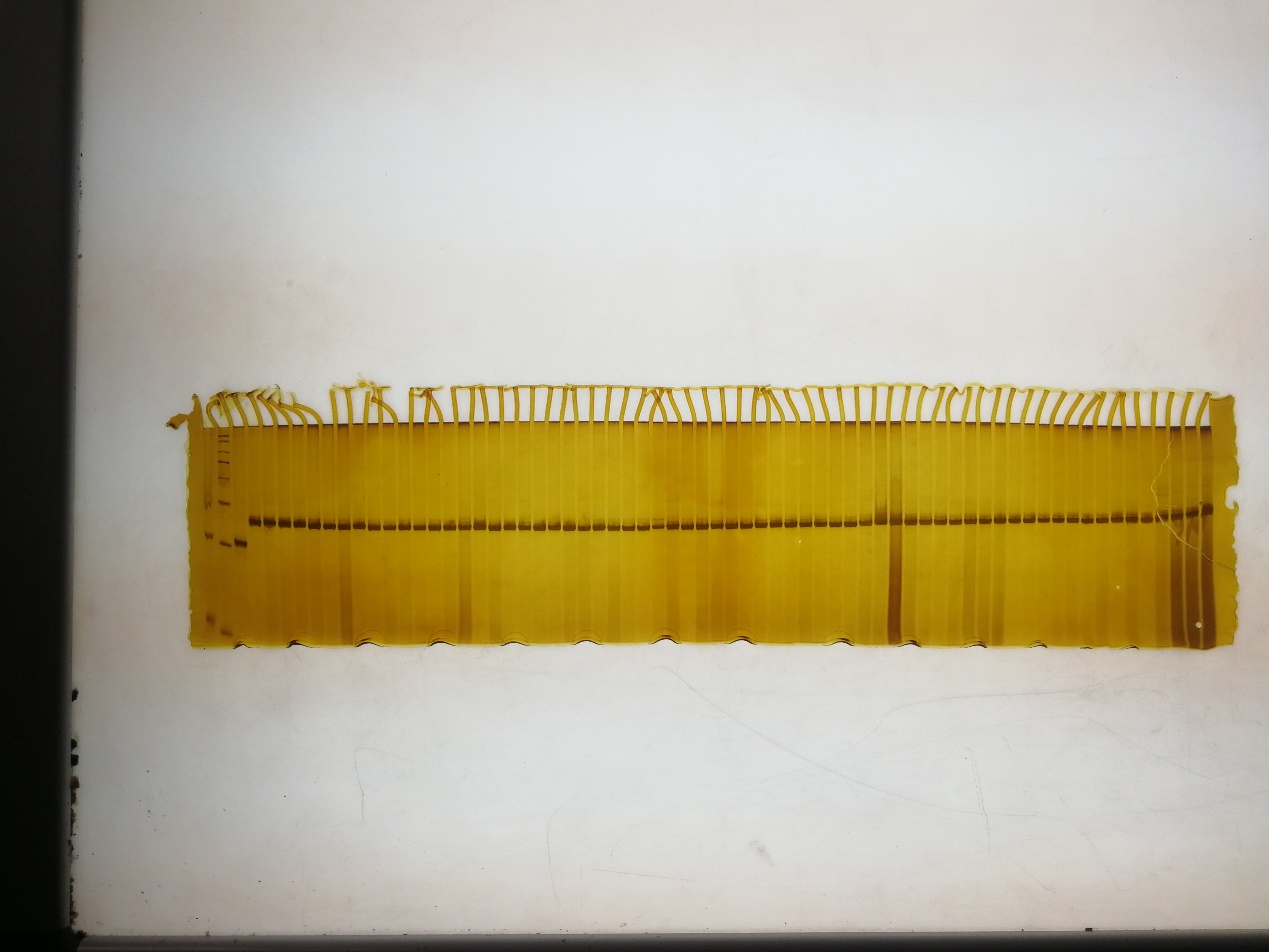


**Figure S2.** The original, full-length gel and blot images of Fig. 6.


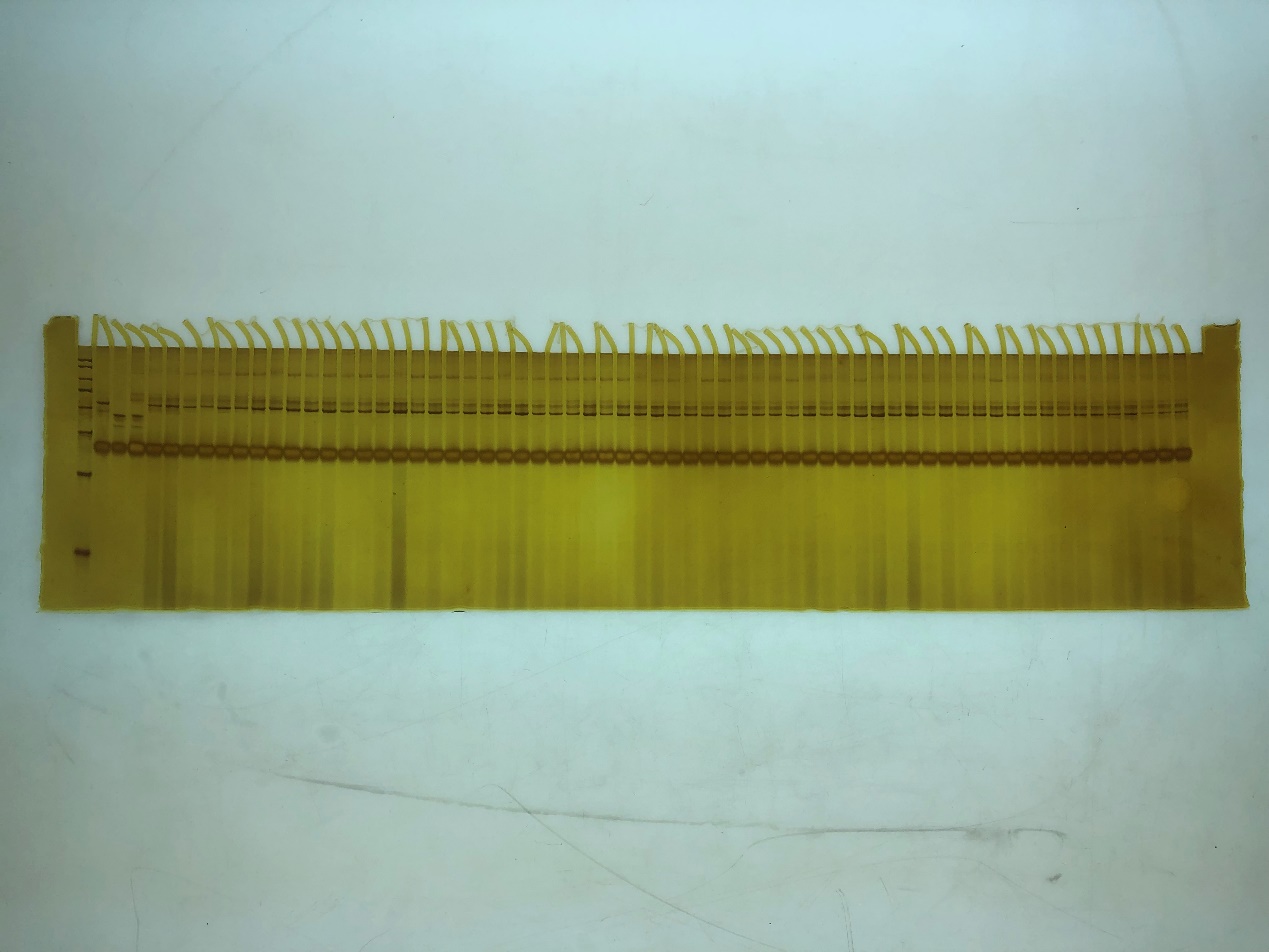


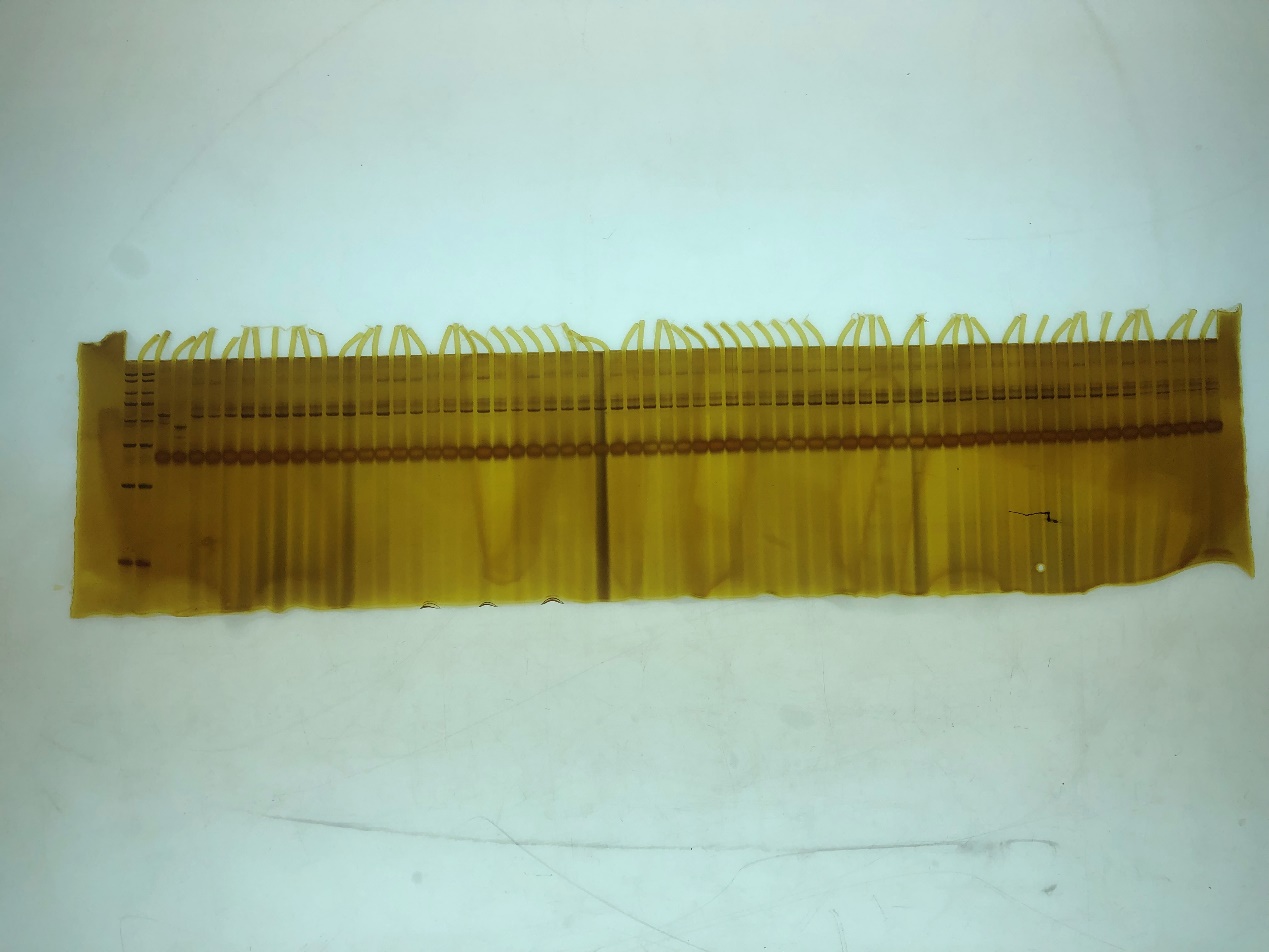


**Figure S3.** The original, full-length gel and blot images of Figure S1.
